# Oral microbial community assembly under the influence of periodontitis

**DOI:** 10.1371/journal.pone.0182259

**Published:** 2017-08-16

**Authors:** Hongju Chen, Shuting Peng, Lin Dai, Quan Zou, Bin Yi, Xianghong Yang, Zhanshan (Sam) Ma

**Affiliations:** 1 College of Mathematics, Honghe University, Mengzi, Yunnan Province, China; 2 Computational Biology and Medical Ecology Lab, State Key Lab of Genetic Resources and Evolution, Kunming Institute of Zoology, Chinese Academy of Sciences, Kunming, China; 3 Faculty of Science, Kunming University of Science and Technology, Kunming, China; 4 Department of Computer Science, Tianjin University, Tianjin China; 5 Oral Medicine Department, Yan’an Hospital of Kunming City, Kunming, Yunnan, China; University of North Texas, UNITED STATES

## Abstract

Several ecological hypotheses (*e*.*g*., specific plaque, non-specific plaque and keystone pathogen) regarding the etiology of periodontitis have been proposed since the 1990s, most of which have been centered on the concept of dysbiosis associated with periodontitis. Nevertheless, none of the existing hypotheses have presented mechanistic interpretations on how and why dysbiosis actually occurs. Hubbell’s neutral theory of biodiversity offers a powerful null model to test hypothesis regarding the mechanism of community assembly and diversity maintenance from the metagenomic sequencing data, which can help to understand the forces that shape the community dynamics such as dysbiosis. Here we reanalyze the dataset from Abusleme *et al*.’s comparative study of the oral microbial communities from periodontitis patients and healthy individuals. Our study demonstrates that 14 out of 61 communities (23%) passed the *neutrality* test, a percentage significantly higher than the previous reported neutrality rate of 1% in human microbiome (Li & Ma 2016, *Scientific Reports*). This suggests that, while the niche selection may play a predominant role in the assembly and diversity maintenance in oral microbiome, the effect of neutral dynamics may not be ignored. However, no statistically significant differences in the neutrality passing rates were detected between the periodontitis and healthy treatments with Fisher’s exact probability test and multiple testing corrections, suggesting that the mechanism of community assembly is robust against disturbances such as periodontitis. In addition, our study confirmed previous finding that periodontitis patients exhibited higher biodiversity. These findings suggest that while periodontitis may significantly change the community composition measured by diversity (*i*.*e*., the exhibition or ‘phenotype’ of community assembly), it does not seem to cause the ‘mutation’ of the ‘genotype” (mechanism) of community assembly. We argue that the ‘phenotypic’ changes explain the observed link (not necessarily causal) between periodontitis and community dysbiosis, which is certainly worthy of further investigation.

## Introduction

The oral cavity is a major portal for food and air with countless microbes exchanging between human body and the environment. At least 6 billions bacteria representing 700 species have been detected in human oral cavity [1]. Several oral infectious diseases such as caries, periodontitis, and endodontic infections are associated with oral microbiome. Besides the pathogens, the oral cavity hosts far more symbiotic bacteria that play a fundamental role in maintaining the oral health. From an ecological perspective, oral microbes exist in the form of microbial community, and oral cavity is an ecosystem. The stability and dynamics of this ecosystem have far reaching influences on our oral health and diseases. Obviously the oral microbial community is subjected to frequent daily physical and biochemical disturbances, but it can maintain its long-term stability in healthy individuals [2].

Periodontitis is a biofilm-induced chronic inflammation of supporting structures of teeth and it may increase the risk for atherosclerosis, diabetes and rheumatoid arthritis [3]. It is characterized by accumulation of bacterial deposits at the gingival margin forming an inflammatory infiltrate, which can lead to the destruction of connective tissue attachment to the tooth, alveolar bone resorption and tooth loss. Significant differences in the oral microbial community composition between periodontitis patients and healthy individuals have been reported [3–6]. Previous studies have revealed that either certain pathogenic bacteria, such as *Porphyromonas gingivalis*, *Treponema denticola* and *Tannerella forsythia*, or the dysbiosis of the normal oral microbial community could play a key role in the etiology of periodontitis [7]. Besides traditional diagnostic criteria such as assessing the attachment of the periodontal tissue to the tooth root and radiographic evidence of bone loss, the presence of *bleeding on probing* (BoP) is also a main clinical indicator of periodontal inflammation and it indicates the risk for periodontitis progression and high level of proinflammatory mediators and destruction markers in gingival exudates [8]. The BoP is likely caused by the formation of plaque at the gingiva due to multiple reasons, such as poor oral hygiene condition and improper tooth brushing.

The culture-dependent method for studying the oral microbiome is neither effective nor efficient though it is often considered as the most reliable method. The Next Generation DNA Sequencing (NGS) technology has leaded to the development and wide adoption of the metagenomics technology, which is culture-independent for investigating the oral microbiome. For example, a pioneering application of the metagenomics technology to the oral micorbiome by Kroes *et al*. detected 59 different species through amplification of 16s-rDNA from a sample of subgingival plaque, which literally doubled the number of species previously found through cultivation (28 species) [9–10]. Another study conducted by Paster *et al*. analyzed 2522 clones from subgingival plaque samples from healthy individuals and patients with refractory periodontitis, adult periodontitis, HIV-infected periodontitis, and acute necrotizing ulcerative gingivitis and detected a number of species only in patients with disease [11]. Aiming to define the normal microbial community of oral cavity, Aas *et al*. sequenced samples from 9 sites of 5 clinically healthy subjects and observed 141 predominant bacterial species inhabiting in these sites, of which over 60% have not been cultivated [11]. Abusleme *et al*. utilized 454-pyrosequencing technology to characterize the subgingival microbial community of 22 patients with chronic periodontitis and compared them with those of healthy individuals, and they observed that several genera exhibited higher proportions in patients with periodontitis, several others exhibited higher proportions in healthy individuals while some exhibited little differences between the health and periodontitis [8]. Their results also indicated that the total bacterial load, species richness and community evenness increased in periodontitis samples [8]. Hong *et al*. studied untreated and root-filled samples with 454-pyrosequencing and found persistent infections displayed similarly diverse bacterial community to the primary infections [12]. Li *et al*. also used 454-pyrosequencing to study the oral microbial community of Chinese patients with aggressive periodontitis [13]; their study suggested that there exists kinship in the phylogenetic architecture of microbial community among patients and their family members. Zhang *et al*. reported that a new virus (TTMV-222) may be associated with inflammation in chronic periodontitis patients using viral metagenomics [14].

Ecological perspectives have already been proposed to interpret the etiologies of several oral diseases including periodontitis since the 1990s, even before the culture-independent metagenomics technology is widely utilized to the study of human oral microbiome (*e*.*g*., Cutler *et al*. 1995, Eriksen and Dimitrov 2003) [15–17]. Although the diversity of microbial community has been found different between patients and healthy individuals in previous studies [3–6], the difference in the mechanism of community assembly and diversity maintenance has not been investigated, to the best of our knowledge. In the present article, we aim to analyze the mechanisms of community assembly and diversity maintenance influenced by periodontitis through the application of Hubbell (2001) neutral theory of biodiversity. This information is potentially of significant importance to the understanding of dysbiosis (*i*.*e*., loss of the microbial community stability) associated with periodontitis.

Traditionally, ecological community is considered being shaped primarily by deterministic forces (factors), such as competition and niche differentiation, but it encounters some difficulties to explain that a number of rare taxa could coexist in very diverse environments [18]. Hubbell’s neutral theory of biodiversity [19] challenged the classic niche theory. It assumes that all individuals within a particular trophic level in a community have the same chances of survival and reproduction independent of their species identity [19], while niche theory assumes that different species occupy different niches, and therefore niche differentiations are necessary for species to coexist. Another important assumption of the neutral theory is that the local community is shaped mainly through stochastic immigration from metacommunity, and the relative abundance of each species in a local community should be similar to its abundance in the metacommunity [20]. Neutral theory that quantifies the neutrality, stochasticity, sampling and dispersal presents a *null* model for testing the mechanism of community assembly and diversity maintenance against species abundance distribution data obtained from practical investigations such as metagenomic sequencing experiments [20–21], as demonstrated in this study.

Neutral theory has three main merits: (*i*) it identifies a minimal but common set of mechanisms explaining observed species abundance distribution (SAD) of an ecological community; (*ii*) it offers an alternative method, potentially more appropriate, to measure biodiversity in than widely used diversity indices; (*iii*) it challenges traditional niche-based theory and stimulates rich debates on the essential topics in community ecology, which should be beneficial to deepening our understanding on the different mechanisms controlling biodiversity [21]. In spite of these advantages, the theory is still relatively less known in microbial ecology, especially in the study of human microbiome [20].

It has long been conjectured that the diversity of oral microbiome is implicated in the etiology of periodontitis. Although a handful of bacteria including *P*. *gingivalis*, *Treponema denticola* and *Tannerella forsythia* have traditionally been considered as causative agents of periodontitis, the recent advances from metagenomic and metatranscriptomic studies have prompted researchers to support an ecological theory based hypothesis. In this hypothesis, periodontitis results from polymicrobial synergy and dysbiosis disturbing the ecologically balanced biofilm associated with periodontal tissue homeostasis [22]. However, this prevalent hypothesis does not explain how and why dysbiosis may occur in the oral microbiome. As mentioned previously, in this article, through testing the *null* model of Hubbell’s neutral theory, we aim to comparatively analyze the assembly and diversity maintenance mechanisms in the oral microbiome influenced by periodontitis. We hope our analysis will help to understand the forces such as periodontitis that shape the community dynamics, including dysbiosis.

## Material and methods

### Dataset description

The dataset we used to test the neutral theory was from a study conducted by Abusleme *et al*. [8].

The study was designed to provide a global-scale view for the microbial community of subgingival and associated inflammation in periodontitis, and adopted balanced samples from both periodontitis patients and healthy controls with appropriate design and sample sizes. Briefly, twenty-two individuals with chronic periodontitis and 10 healthy individuals were recruited in this study. Subjects with periodontitis were sampled at two non-adjacent tooth sites that differ in the presence of BoP (Bleeding on Probing). Healthy individuals were sampled at two subgingival plaque sites without BoP. DNA was extracted and amplified for the V1-V2 hyper-variable regions of 16S rRNA gene and then sequenced using 454 titanium chemistry. Forty-four samples from the individuals with periodontitis and 17 samples from the healthy individuals were sequenced successfully. The raw data is available at the Short Reads Archive (Accession number SRA051864).

We downloaded the raw data from the Short Reads Archive and recalculated the OTU (Operational Taxonomic Units) Tables using Mothur software [23]. After quality control, the dataset included 153286 sequences with an average of 2512 (range 591–10130) sequences per sample. We picked OTUs at 97% similarity level (the most commonly used cutoff for the assignment of 16S sequences at species level) and generated 673 OTUs in total with a range of 29–165 OTUs per sample. The recalculated OUT tables were then divided into 3 treatments based on original study of Abusleme *et al*. [8]: BoP treatment (the samples from sites with BoP in subjects with periodontitis), Non-BoP treatment (the samples from sites without BoP in subjects with periodontitis), and control treatment (the samples from healthy individuals).

### The computational procedure for testing the neutral theory

To test the neutral theory null model, we adopted two most widely used sampling formulae (probability distributions). The first one was originally developed in population genetics by Ewens [24], and later introduced into ecology by Hubbell (2001) for calculating the likelihood of the presence of an ecological community consisting of *S* species with abundance of *n*_*1*_, *n*_*2*…_*n*_*s*_ and measuring its consistency with the prediction of neutral theory. It has the following form:

$$P_{r}(n_{1},n_{2}\ldots \ldots n_{s}|\theta ,J)=\frac{J!\theta^{s}}{1^{\phi_{1}{}^{}}2^{\phi_{2}{}^{}}\ldots J^{\phi_{J}{}^{}}\phi_{1}!\phi_{2}!\ldots \phi_{J}!\prod_{K=1}^{J}\left(\theta +K-1\right)}$$ (1)

where, *S* represents for the number of species, *J* for the number of individuals, *θ* is the fundamental parameter of biodiversity parameter, *n*_*i*_ is the abundance of species *i*, and *φ*_*a*_ is the number of species with abundance *a*. Note that Ewens formula does not take into account the effect of dispersal limitation, assuming that the immigration rate *m* = 1.

The biodiversity parameter *θ* is defined as:

$$\theta =J_{M}\frac{v}{1-v}$$ (2)

Where *J*_*M*_ is the number of individuals in the metacommunity and *v* is the per capita speciation rate. The parameter *θ* can be estimated *via* maximum likelihood estimation (MLE) method [25][26].

The second formula was developed by Etienne [25] and is considered as one of the best performed sampling approach to test the neutral theory. It incorporated the dispersal limitation and should be utilized if dispersal limitation is in effect (*i*.*e*., *m*<1). The application of this formula for microbiome dataset was demonstrated in Li & Ma’s study [20]. Etienne (2005)[25] sampling formula considered the dispersal limitation, which is measured by the immigration probability (*m*), and *m* is defined as:

$$m=\frac{I}{I+J-1}$$ (3)

where *I* is the number of immigrants that are searching for vacant spots in the local community (*i*.*e*., competing with the local individuals), and *J* is the number of individuals.

Etienne (2005)[25] sampling formula is defined as:

$$P_{r}(D|\theta ,m,J)=\frac{J!}{\prod_{i=1}^{S}n_{i}\prod_{j=1}^{J}\phi_{j}!}\frac{\theta^{S}}{{\left(I\right)}_{J}}\sum_{A=S}^{J}K(D,A)\frac{I^{A}}{{\left(\theta \right)}_{A}}$$ (4)

where *K*(*D*, *A*) is further defined as

$$K(D,A)=\sum_{\left\{a_{1},a_{2}\ldots .a_{S}|\sum_{i=1}^{S}a_{i}=A\right\}}\prod_{i=1}^{S}\frac{{\bar{S}}_{\left(n_{i},a_{i}\right)}{\bar{S}}_{\left(a_{1},1\right)}}{{\bar{S}}_{\left(n_{i},1\right)}}.$$ (5)

We compare the *log-likelihoods* computed with Ewens formula and Etienne formulae to determine which of the formulae is more appropriate for the oral microbiome dataset of this study. We further compare the performance (*D*) of both formulae to evaluate the possible effect of dispersal limitation using the following *log-likelihood ratio test*,

$$D=-2\ln(\frac{L_{0}}{L_{1}})=-2(\ln(L_{0})-\ln(L_{1}))$$ (6)

where, *D* is the deviation that is twice the difference between the log-likelihoods of the two formulae, *L*_*0*_ and *L*_*1*_ are the log-likelihood of the null model and alternative model, respectively. The *p*-value calculated follows a Chi-Squared distribution with the degree of freedom of one.

After the comparison, we adopted an *exact neutrality test* method [26] to test the neutrality of samples, *i*.*e*., whether or not the sample distribution of the observed community satisfies with the prediction of the neutral theory null model. Specifically, the neutrality exact test compares the probability (the likelihood) of the realized or observed configuration (community species abundance distribution) with the probabilities of the artificially simulated configurations. It adopts a mixture test strategy of Monte Carlo significance test and the parametric bootstrap. The test is ‘exact’ since type I error can be exactly specified [26].

Computationally, we utilized a standard *R*-package UNTB (Unified Neutral Theory of Biodiversity) (freely available at: https://cran.r-project.org/web/packages/untb/index.html) that implemented the MLE method for estimating the parameters of both Ewens and Etienne formulae in terms of the observed samples. The major computational steps can be outlined as the following four steps: (*i*) For each community sample, 100 artificial communities (datasets) are simulated using the parameters (*θ*, *I*, *J*) estimated with the observed samples. (*ii*) Etienne formula is used to calculate the likelihood for each artificial dataset, namely *P*_*s*_. (*iii*) The mean value of the likelihoods (*P*_*s*_) of 100 artificial datasets for each sample and the likelihood (*P*_*0*_) of the corresponding observed sample are compared using the *log-likelihood ratio* test, under the null hypothesis that there is no significant difference between *P*_*0*_ and *P*_*s*._ (*iv*) If the *p-*value from the *log-likelihood ratio* test exceeds 0.05, we conclude that the neutrality of the community under testing cannot be rejected, and that the observed species abundance distribution (SAD) is consistent with the prediction from the neutral theory, *i*.*e*., passing the neutrality test. In addition, as mentioned previously, the *log-likelihood ratio* test was performed with both Ewens and Etienne formulae, respectively to compare their performance.

### The statistical tests for further verifying the neutrality-test results

***Multiple testing correction*** or multiple testing for short (also know as multiple comparisons or multiplicity test) is designed to correct the increased chance of incorrectly rejecting the null hypothesis (*i*.*e*., false positives or Type-I errors) with the increased number of (multiple) tests (Nobel 2009) [27]. We utilize R-package EMA (Easy Microarray Data Analysis) (https://CRAN.R-project.org/package=EMA) maintained by Servant *et al*. (2016) [28]. EMA implemented the statistical procedures for multiple testing developed by Benjamini and Hochberg (1995) [29].

Fisher’s exact probability test is designed to compare the frequency of occurrences (observations) in a fourfold table setting when the numbers are too small to use the Chi-square test. We use Fisher’s exact probability test to determine if there are pair-wise differences between the treatments in their passing rates of the neutrality test. We used the *R*-function (fisher.test) from the standard R-package (stats) (http://stat.ethz.ch/R-manual/R-patched/library/stats/html/fisher.test.html) to conduct the Fisher’s exact test in this study.

## Results and discussion

### Comparison of Ewens and Etienne sampling formulae

For each sample, we tested its neutrality with both Ewens and Etienne sampling formulae, and detailed results were provided in the online Supplementary S1 Table and S2 Table, respectively. The log-likelihood ratio test was used to compare the performance of two formulae in testing the neutral theory with the datasets of the three treatments (Periodontitis with BoP, Periodontitis non-BoP, and the Healthy) respectively.

S3 Table listed the detailed results from comparing Ewens and Etienne sampling formulae. There were no significant differences between Ewens and Etienne formulae in terms of the log-likelihood (*p*>0.05) in all samples we tested (S3 Table). Both methods performed equally well, and Etienne formula was chosen to test the neutrality in this study, given the latter has an advantage for considering dispersal limitation.

### Testing the neutral theory with Etienne neutrality exact test

A total of 6100 artificial datasets (simulated communities) corresponding to 61 observed community samples (each was matched with 100 artificially simulated communities) were generated using the parameters estimated from the observed community samples. For each sample, the likelihood (*P*_*0*_) and the average likelihood (*P*_*s*_) of 100 artificial datasets were compared using the *log-likelihood ratio test*. In general, 22.95% (14 out of 61) of all community samples passed the neutrality exact test. The parameters and test results of the community samples that passed the neutrality test were listed in Table 1 below, and the results of all samples were listed in the online Supplementary S1 & S2 Tables. As mentioned previously, S3 Table exhibited the results from comparing the two sampling formulae in testing the neutrality.

**Table 1:** The 14 communities that passed the neutrality exact tests with both Ewens and Etienne sampling formulae (with 100 artificially simulated communities)*.

Treatment & Number of Samples	Ewens sampling formula						Etienne sampling formula			
	*ID*	*J*	*S*	*θ*	*p*-value	*p*-value *Adjusted*	*θ*	*m*	*p*-value	*p*-value *Adjusted*
Healthy (17)	25H2	537	37	8.856	0.2808	0.3114	8.815	0.99710	0.2383	0.2692
	28H2	687	75	21.250	0.2363	0.2669	21.273	0.99998	0.3319	0.3681
	28H1	1030	86	22.147	0.0472	0.0587	22.127	0.99995	0.0417	0.0530
BoP (22)	12PB	825	85	23.587	0.0775	0.0928	23.597	0.99973	0.1459	0.1679
	17PB	1421	106	26.333	0.4256	0.4476	26.315	0.99875	0.4471	0.4785
	3PB	2089	122	28.116	0.2142	0.2465	28.112	0.99982	0.6374	0.6480
	7PB	3423	165	36.043	0.0038	0.0071	36.898	0.80972	0.0519	0.0647
	9PB	3600	130	26.284	0.4565	0.4719	26.320	0.99974	0.1367	0.1635
Non-BoP (22)	13PnB	1192	75	17.616	0.0766	0.0928	17.637	0.99989	0.1399	0.1641
	18PnB	1147	99	25.812	0.2872	0.3129	25.834	0.99988	0.5354	0.5631
	1PnB	1453	106	26.144	0.4160	0.4452	26.171	0.99993	0.7026	0.7026
	21PnB	591	95	31.763	0.1501	0.1761	31.743	0.99995	0.1298	0.1584
	23PnB	3569	131	26.596	0.5873	0.5873	26.571	0.99562	0.4159	0.4531
	5PnB	1824	126	30.571	0.4855	0.4936	30.621	0.99991	0.6068	0.6274

*J: the total number of reads in the sample, *S*: the number of species in the sample, *θ*: fundamental biodiversity number, *m*: migration probability, *p*-value: calculated from the log-likelihood ratio test, and *p*-value adjusted: the *p*-value adjusted with multiple correlation correction procedure. In the cases of sample 28H1 and 7PB (shaded in grey), the significance for passing the neutrality test slightly increased after adjusting the *p*-value with multiple testing correction.

Listed in Table 1 are the columns of sample ID, for each formula, the total number of reads (individuals) in the sample (*J*), the number of species (*S*), the fundamental biodiversity number (*θ)*, *p-*value from the log-likelihood ratio test, and adjusted *p*-value with multiple testing correction.

It is noted that 100 artificially simulated communities have been the default choice in testing the neutral theory with the *UNTB* R package, mentioned previously. In the peer-review process, an anonymous expert reviewer suggested to increase the number of simulations to 1000 times or more. We concur with the expert since more simulations should help to relieve the random differences from stochastic simulations. The results from 1000 artificial communities were listed in the online Supplementary S4 Table. Table 2 below listed the samples that passed the neutrality test utilizing Etienne formula, which we prefer to Ewens formula as explained previously, with 1000 artificial communities simulated for each actually observed community. That is, the distinction between Tables 1 and 2 lies in the numbers of artificial communities (100 *vs*. 1000), and only testing with Etienne formula is performed in the latter.

**Table 2:** The 13 communities that passed the neutrality exact tests with Etienne sampling formula with 1000 artificially simulated communities*.

Treatment & Number of Samples	*ID*	*J*	*S*	*θ*	*m*	*p-*value	*p-*value *Adjusted*
Healthy (17)	25H2	537	37	8.815	0.9971	0.1300	0.1496
	28H2	687	75	21.273	0.99998	0.4054	0.4191
BoP (22)	12PB	825	85	23.597	0.99973	0.1173	0.1403
	17PB	1421	106	26.315	0.99875	0.2706	0.3001
	18PB	1116	128	38.435	0.8351	0.0664	0.0810
	3PB	2089	122	28.112	0.99982	0.287	0.3109
	9PB	3600	130	26.32	0.99974	0.0551	0.0686
Non-BoP (22)	13PnB	1192	75	17.637	0.99989	0.1486	0.1679
	18PnB	1147	99	25.834	0.99988	0.5584	0.5677
	1PnB	1453	106	26.171	0.99993	0.2905	0.3109
	21PnB	591	95	31.743	0.99995	0.1269	0.1489
	23PnB	3569	131	26.571	0.99562	0.9268	0.9268
	5PnB	1824	126	30.621	0.99991	0.4017	0.4191

*J: the total number of reads in the sample, *S*: the number of species in the sample, *θ*: fundamental biodiversity number, *m*: migration probability, *p*-value: calculated from the log-likelihood ratio test, and *p*-value adjusted: the *p*-value adjusted with multiple testing correction procedure.

Another suggestion from the reviewer is to adjust the *p*-value with *multiple testing correction* procedure. Indeed, we adopted this rather meaningful adjustment in all of the results reported in this study, except for the results in S3 Table for comparing Ewens and Etienne sampling formulae, where log-likelihoods were compared and therefore the adjustment is unnecessary. The 1000 times of artificial communities and the multiple testing correction did lead to the “flip-flop” of the testing results of only three samples (28H1, 7PB, and 18PB), although the flip-flop did not change the general conclusions of the neutrality test (see Table 3). Specifically, the “flip-flop” of the three samples led to only one net reduction of the samples that pass the neutrality test, when the number of artificially simulated communities increased from 100 to 1000.

**Table 3:** The “flip-flop” of the neutrality test results with Etienne formula with 100/1000 artificial communities*.

Treatment	Ewens sampling formula (with 100 artificial communities)						Etienne sampling formula (with 100 artificial communities)				Etienne Formula (1000 communities)	
	*ID*	*J*	*S*	*θ*	*p-*value	*p-*value *Adjusted*	*θ*	*m*	*p-*value	*p-*value *Adjusted*	*p-*value	*p-*value *Adjusted*
Healthy	28H1	1030	86	22.147	0.0472	0.0587	22.127	0.99995	0.0417	0.0530	0.0243	0.0322
BoP	7PB	3423	165	36.043	0.0038	0.0071	36.898	0.80972	0.0519	0.0647	0.0217	0.0316
	18PB	1116	128	37.107	0.0216	0.0293	38.435	0.8351	0.0311	0.0404	0.0664	0.0810

*J: the total number of reads in the sample, *S*: the number of species in the sample, *θ*: fundamental biodiversity number, *m*: migration probability, *p*-value: calculated from the log-likelihood ratio test, and *p*-value adjusted: the *p*-value adjusted with multiple testing correction procedure. The “flip-flop” of the three samples led to net one reduction of the samples that pass the neutrality test, when the number of artificially simulated communities increased from 100 to 1000. All other 58 samples did not experience any flip-flop when the simulations were increased 10 times.

**Fig 1:**
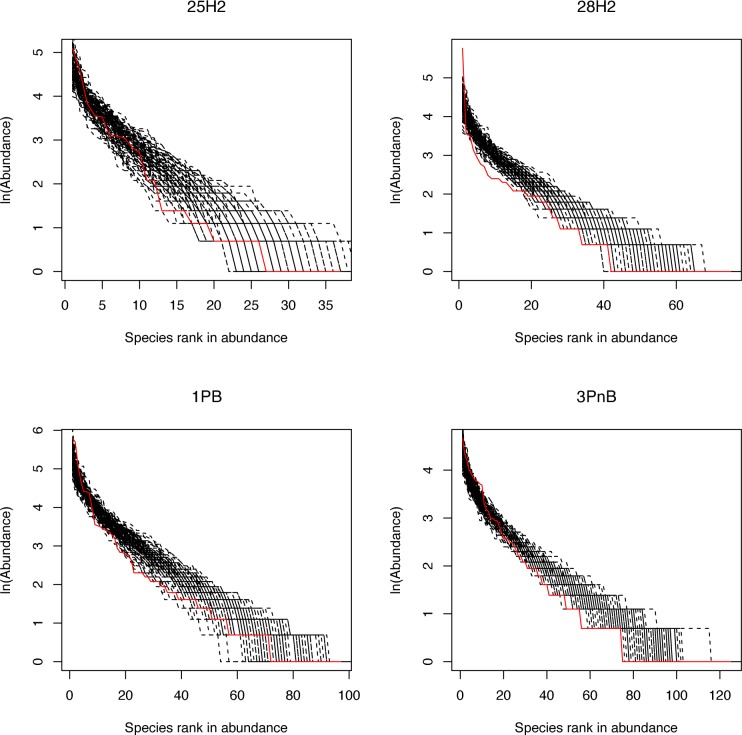
The rank abundance curves of four demonstrative samples that successfully pass the neutrality test. The four samples were selected from four different individual subjects among 3 treatments: 1 from the BoP treatment (Subject ID: 1PB), 1 from the Non-BoP (Subject ID: 3PnB), and 2 from the healthy treatment (Subject ID: 25H2, 28H2), the solid red line represents for the observed data and the black dash lines for the simulated datasets. The *X*-axis is the species rank order in abundance and *Y*-axis is the abundance of each species in natural logarithm.

From Tables 1, 2 and 3, supplemented by S1–S4 Tables, we summarize the following findings regarding the testing of the neutral theory with Etienne formula. In the periodontitis BoP treatment, 22.73% (5/22) of the samples satisfied the neutral prediction, in the periodontitis non-BoP treatment, 27.27% (6/22) of the samples passed the neutrality exact test, and in the healthy (control) treatment, 17.65% (3/17) of the samples passed the test. Although the passing rates in the periodontitis treatments (BoP and non-BoP) are apparently higher than in the healthy treatment, rigorous testing with Fisher’s exact probability test suggested that there are not any significant differences among the three treatments. The *p*-value of Fisher’s exact test for the difference between the healthy and periodontitis BoP treatments is *p* = 1, that for the difference between the healthy and periodontitis Non-BoP treatments is *p* = 0.704, and *p* = 1 between periodontitis BoP and periodontitis Non-BoP treatments. This result suggests that periodontitis does not seem to significantly affect the assembly mechanism of oral microbial communities, and the observed differences in passing rates of the neutrality test should be due to random effects. In other words, whether or not the assembly of a community is driven by the stochastic neutral dynamics or deterministic niche forces is independent of the occurrence of periodontitis. This also means that the nature or type of the community assembly mechanism in the oral microbiome is not significantly impacted by periodontitis disease. Fig 1 shows the curves of 4 samples out of 61 samples that satisfy the prediction of the neutral theory model with Etienne neutrality exact test.

### Comparison of the fundamental diversity numbers (*θ*)

**Fig 2:**
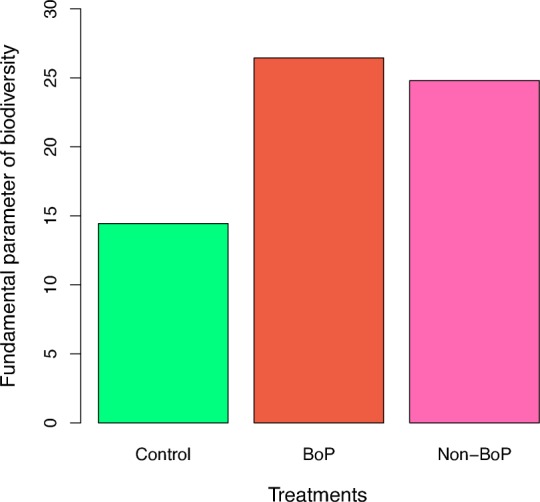
The average values of the fundamental diversity parameter (*θ*) in the three treatments obtained with Etienne sampling formula: significant differences between control (healthy) and BoP (periodontitis with BoP), as well as between control (healthy) and Non-BoP (periodontitis without BoP) were detected (The Bonferroni pair-wise comparison with *p*<0.01).

For each of the three treatments (the healthy, periodontitis Non-BoP and periodontitis NoP), the average biodiversity parameter (*θ*) was calculated and displayed in Fig 2. The analysis of variance was conducted to compare the average *θ* value of the 3 treatments and significant differences were detected among the 3 treatments (*p-*value<0.01). Bonferroni pair-wise comparison was further performed. Both BoP treatment and Non-BoP treatment showed significantly higher *θ* value than the healthy (control) treatment (*p-*value<*0*.*01*). But there was not any significant difference between BoP treatment and Non-BoP treatment in terms of the *θ* value.

Compared with the previous finding about the lack of significant differences in the passing rates of the neutrality tests, the significant differences in the average biodiversity parameter (*θ*) prompt us to propose the following hypothesis for future further study. That is, while periodontitis may significantly change the community composition (exhibited by the change of biodiversity), it may not significantly alter the nature (*i*.*e*., niche or neutral) of the community assembly mechanism.

We postulate that dramatic changes in community composition are likely to be associated with dysbiosis, which has been linked to the occurrence of periodontitis. Our previous findings support this hypothesis, but our findings do not provide evidence to establish a causal relationship. We do not know whether dysbiosis is the cause or consequence of periodontitis.

## Conclusions

By comparing Ewens and Etienne formulae, performing the neutrality test with Etienne formula, and conducting further tests with multiple testing correction and Fisher’s exact probability test, as well as comparing the fundamental biodiversity number among three treatments, we obtained the following conclusions and postulations for further study. (*i*) While deterministic niche forces are predominant in driving the oral community assembly, the role of stochastic neutral dynamics cannot be ignored (in our case, approximately 23% (14 out of 61) of community samples satisfied with the neutral theory). (*ii*) Periodontitis does not seem to alter the assembly mechanism of oral microbial communities. (*iii*) However, periodontitis may indeed change community composition or diversity of the oral microbiome. Dramatic changes in community compositions (diversity) may lead to dysbiosis, which may be linked to periodontitis.

Using an analogy with the genotype-phenotype relationship in modern genetics to express our findings, periodontitis disease does not seem to cause mutation in the ‘genotype’ (mechanism) of community assembly, but may impact the ‘phenotype’ (exhibition of community assembly or community composition measured in the fundamental biodiversity number). The *dysbiosis* associated with periodontitis may simply be the sufficiently dramatic phenotypic changes, but the direction of the link between dysbiosis and disease is yet to determine by future studies.

## Discussion

In early days, oral diseases were attributed to a handful of specific pathogens in the oral microbial community. The idea was referred to as “*specific plaque hypothesis*” proposed by Loesche [30]. Later, the updated “*non-specific plaque hypothesis*” was proposed, and the updated hypothesis stated that all bacteria in a plaque contribute to the pathogenicity of the microbial community *via* colonization, evasion or provocation of inflammation and tissue destruction [31]. In fact, the general idea had already been proposed in the “*traditional non-specific plaque hypothesis*” as early as in the 19^th^ century [32]. Since people at that time could not identify specific pathogens, so they treated oral diseases by removing the whole plaque. The updated idea was more like an ecological idea, which took into account the virulence of different species in the microbial community. In 1994, “*ecological plaque hypothesis*” was proposed [33]. This theory integrating the core idea of early hypotheses stated that diseases are caused by the imbalance of the whole microbial community, which is essentially the concept of *dysbiosis* in more recent literatures. The imbalance may result from ecological stress (disturbances) and can lead to the occurrence of disease-related microorganisms. The latest hypothesis “*keystone pathogen hypothesis*” [7] suggested that some low-abundance pathogens may have the ability to tip the balance between normally benign (healthy) microbiota and dysbiotic one and cause diseases ultimately. The hypothesis apparently took inspirations from macro-ecology. The effect of these keystone species is similar to the top predators (such as tigers) in an ecosystem, who usually has a discretionally large effect on the ecosystem but with a tiny relative abundance. The keystone species therefore may play a critical role in maintaining the community structure. The keystone pathogen theory appears to offer a plausible explanation to the significant difference in the composition of microbial community between patients with diseases and healthy individuals. Nevertheless, there is a problem with this hypothesis. In macro-ecology, keystone species can be any species at any trophic level. Similarly, there may be many keystone species, not necessarily pathogens, in a microbial community that may have significantly large influence incommensurable with their relative abundances. Indeed, keystone species may be the natural enemies of pathogens such as bacteriophages.

Abulsleme *et al*. found increased total bacterial load and richness as well as increased evenness in patients with periodontitis [8]. One objective of our study is to explore whether the biodiversity of oral microbial community would be changed by periodontitis using the fundamental biodiversity parameter (*θ*) of the neutral theory model, which is considered to be more reliable than the traditional diversity indexes such as Shannon index and Simpson index, given that *θ* is estimated based on the full information from SAD of a community. Our results confirmed Abulsleme *et al*. previous finding: that the periodontitis treatment (both BoP and Non-Bop treatments) exhibited significantly higher diversity than the control treatment. The finding appears to support the holistic *ecological plaque hypothesis*.

Understanding the mechanism of community assembly and diversity maintenance through approaches such as testing the neutral null model has far-reaching ramifications to the understanding of oral diseases such as periodontitis. This is because the acceptance or rejection of the previously mentioned hypotheses on the oral diseases ultimately depends on the deep understanding of the oral microbial communities, not only their structures, but also their stabilities and dynamics. The latter should be more important. Unfortunately, direct studies of community dynamics are far more difficult than investigating the community structure. This is especially true in biomedical research of the human microbiome because manipulative observations are limited by ethical considerations. It is for this reason that most existing studies of the human microbiome are still focused on the study of community structure (composition) through sequencing the community samples, which is neither intrusive and nor unethical. Neutral theory we adopted in this study offers an ideal ecological tool to decipher the mechanistic information about the formation of community structure (*i*.*e*., more formally the mechanism of community assembly and diversity maintenance) from the OTU tables [*i*.*e*., more formally the species abundance distribution (SAD) data]. The test of neutral theory null model is essentially a fit of a special SAD model, a zero-sum multinomial distribution model, which can be derived from the basic assumptions of the neutral theory.

We postulated that the link between periodontitis and dysbiosis might be explained by the observed differences in the fundamental biodiversity numbers between the healthy and periodontitis treatments. For the previously discussed reason (*i*.*e*., manipulative experiments routinely conducted in natural ecological communities such as forest and lakes are infeasible for the human microbiome), we cannot determine the nature of this link—whether or not it is causal. This limitation may exist in other ecological studies of microbiome-associated diseases. Indeed, it may be a fundamental limit that makes the medical ecology of the human microbiome different from microbial ecology [34].

## Supporting information

S1 Table The result of the neutrality test using Ewen’s formula (Full results for all samples). (DOC)

S2 Table The result of the neutrality test using Etienne’s formula (Full results for all samples). (DOC)

S3 Table The detailed results from the comparison of Ewens and Etienne sampling formulae. (DOC)

S4 Table The result of the neutrality test using Etienne formula with 1000 times of simulations. (DOC)
