# Supplementary material for: Oral microbial community assembly under the influence of periodontitis
Source: PLoS One. 2017 Aug 16;12(8):e0182259. doi: 10.1371/journal.pone.0182259 (PMC5558961; doi:10.1371/journal.pone.0182259)
Supplement: S1 Table — (DOC) [file pone.0182259.s001.doc]

**S1 Table**. The result of the neutrality test using Ewen’s formula (Full results for all samples)*

| Treatment | *ID* | *J* | *S* | *θ* | log(L_0_) | log(L_1_) | *q-value* | *p-value* | *p-value*  *adjusted* |
| --- | --- | --- | --- | --- | --- | --- | --- | --- | --- |
| Control | 24H2 | 775 | 38 | 8.231 | -48.425 | -52.651 | 8.452 | 0.0036 | 0.0070 |
|  | 25H1 | 890 | 29 | 5.617 | -42.105 | -50.813 | 17.415 | 0.0000 | 0.0001 |
|  | 25H2* | 537 | 37 | 8.856 | -44.021 | -44.603 | 1.163 | 0.2808 | 0.3114 |
|  | 26H1 | 917 | 53 | 12.092 | -58.626 | -60.922 | 4.590 | 0.0322 | 0.0416 |
|  | 27H1 | 741 | 75 | 20.653 | -59.763 | -55.181 | 9.164 | 0.0025 | 0.0050 |
|  | 27H2 | 891 | 124 | 38.921 | -63.338 | -57.362 | 11.952 | 0.0005 | 0.0013 |
|  | 28H1 | 1030 | 86 | 22.147 | -64.608 | -66.577 | 3.939 | 0.0472 | 0.0587 |
|  | 28H2* | 687 | 75 | 21.250 | -53.352 | -52.651 | 1.403 | 0.2363 | 0.2669 |
|  | 29H1 | 6126 | 123 | 21.693 | -142.952 | -158.780 | 31.656 | 0.0000 | 0.0000 |
|  | 29H2 | 5567 | 66 | 10.425 | -101.008 | -125.872 | 49.730 | 0.0000 | 0.0000 |
|  | 30H1 | 5294 | 68 | 10.909 | -107.553 | -125.470 | 35.835 | 0.0000 | 0.0000 |
|  | 30H2 | 4922 | 71 | 11.656 | -100.168 | -122.945 | 45.554 | 0.0000 | 0.0000 |
|  | 31H1 | 6810 | 77 | 12.071 | -109.646 | -141.761 | 64.229 | 0.0000 | 0.0000 |
|  | 31H2 | 10130 | 54 | 7.407 | -91.383 | -135.914 | 89.061 | 0.0000 | 0.0000 |
|  | 32H1 | 8670 | 65 | 9.453 | -98.908 | -143.776 | 89.735 | 0.0000 | 0.0000 |
|  | 33H1 | 8366 | 75 | 11.267 | -111.284 | -148.781 | 74.993 | 0.0000 | 0.0000 |
|  | 33H2 | 4575 | 74 | 12.434 | -109.390 | -123.950 | 29.121 | 0.0000 | 0.0000 |
| BoP | 10PB | 3159 | 137 | 29.056 | -117.985 | -121.024 | 6.077 | 0.0137 | 0.0194 |
|  | 11PB | 2764 | 92 | 18.190 | -91.589 | -108.174 | 33.171 | 0.0000 | 0.0000 |
|  | 12PB* | 825 | 85 | 23.587 | -60.076 | -58.518 | 3.116 | 0.0775 | 0.0928 |
|  | 13PB | 1210 | 125 | 34.811 | -73.991 | -70.834 | 6.316 | 0.0120 | 0.0187 |
|  | 14PB | 3606 | 76 | 13.503 | -89.392 | -113.892 | 48.999 | 0.0000 | 0.0000 |
|  | 15PB | 3211 | 86 | 16.137 | -108.846 | -112.691 | 7.691 | 0.0056 | 0.0092 |
|  | 16PB | 1176 | 82 | 19.898 | -64.285 | -71.445 | 14.320 | 0.0002 | 0.0004 |
|  | 17PB* | 1421 | 106 | 26.333 | -78.995 | -78.677 | 0.635 | 0.4256 | 0.4476 |
|  | 18PB | 1116 | 128 | 37.107 | -69.510 | -66.873 | 5.274 | 0.0216 | 0.0293 |
|  | 19PB | 1025 | 122 | 35.880 | -69.055 | -63.739 | 10.632 | 0.0011 | 0.0024 |
|  | 1PB | 1788 | 97 | 21.849 | -84.899 | -88.821 | 7.843 | 0.0051 | 0.0092 |
|  | 20PB | 1247 | 157 | 47.296 | -79.696 | -69.705 | 19.982 | 0.0000 | 0.0000 |
|  | 21PB | 1487 | 101 | 24.344 | -76.008 | -81.043 | 10.072 | 0.0015 | 0.0032 |
|  | 23PB | 947 | 100 | 28.041 | -65.227 | -62.252 | 5.949 | 0.0147 | 0.0204 |
|  | 2PB | 1205 | 58 | 12.573 | -60.289 | -69.172 | 17.768 | 0.0000 | 0.0001 |
|  | 3PB* | 2089 | 122 | 28.116 | -97.807 | -97.035 | 1.543 | 0.2142 | 0.2465 |
|  | 4PB | 1157 | 109 | 29.326 | -74.658 | -70.259 | 8.799 | 0.0030 | 0.0059 |
|  | 5PB | 1132 | 80 | 19.492 | -66.763 | -69.870 | 6.213 | 0.0127 | 0.0192 |
|  | 6PB | 2334 | 80 | 15.916 | -78.678 | -98.006 | 38.657 | 0.0000 | 0.0000 |
|  | 7PB | 3423 | 165 | 36.043 | -123.394 | -127.578 | 8.368 | 0.0038 | 0.0071 |
|  | 8PB | 4334 | 153 | 30.781 | -131.381 | -141.395 | 20.029 | 0.0000 | 0.0000 |
|  | 9PB* | 3600 | 130 | 26.284 | -127.905 | -128.182 | 0.555 | 0.4565 | 0.4719 |
| Non-BoP | 10PnB | 1532 | 138 | 36.589 | -84.990 | -81.142 | 7.695 | 0.0055 | 0.0092 |
|  | 11PnB | 1929 | 91 | 19.698 | -89.606 | -92.015 | 4.819 | 0.0281 | 0.0373 |
|  | 12PnB | 2451 | 89 | 17.979 | -94.224 | -101.631 | 14.815 | 0.0001 | 0.0003 |
|  | 13PnB* | 1192 | 75 | 17.616 | -73.192 | -71.624 | 3.136 | 0.0766 | 0.0928 |
|  | 14PnB | 3505 | 50 | 8.160 | -81.460 | -96.841 | 30.761 | 0.0000 | 0.0000 |
|  | 15PnB | 3238 | 60 | 10.348 | -83.405 | -103.375 | 39.941 | 0.0000 | 0.0000 |
|  | 16PnB | 1714 | 58 | 11.468 | -63.165 | -80.410 | 34.490 | 0.0000 | 0.0000 |
|  | 17PnB | 1745 | 129 | 31.988 | -91.228 | -88.156 | 6.143 | 0.0132 | 0.0192 |
|  | 18PnB* | 1147 | 99 | 25.812 | -71.087 | -70.521 | 1.133 | 0.2872 | 0.3129 |
|  | 19PnB | 1271 | 85 | 20.364 | -77.750 | -74.665 | 6.170 | 0.0130 | 0.0192 |
|  | 1PnB | 1453 | 106 | 26.144 | -80.265 | -80.596 | 0.662 | 0.4160 | 0.4452 |
|  | 20PnB | 1134 | 122 | 34.494 | -74.453 | -67.846 | 13.213 | 0.0003 | 0.0007 |
|  | 21PnB* | 591 | 95 | 31.763 | -47.409 | -46.374 | 2.071 | 0.1501 | 0.1761 |
|  | 23PnB* | 3569 | 131 | 26.596 | -127.774 | -127.626 | 0.295 | 0.5873 | 0.5873 |
|  | 2PnB | 2062 | 83 | 17.207 | -86.945 | -93.606 | 13.322 | 0.0003 | 0.0007 |
|  | 3PnB | 1077 | 125 | 36.402 | -71.264 | -65.917 | 10.695 | 0.0011 | 0.0024 |
|  | 4PnB | 833 | 59 | 14.343 | -62.011 | -58.605 | 6.812 | 0.0091 | 0.0145 |
|  | 5PnB* | 1824 | 126 | 30.571 | -90.961 | -90.718 | 0.486 | 0.4855 | 0.4936 |
|  | 6PnB | 4172 | 118 | 22.469 | -115.159 | -133.872 | 37.427 | 0.0000 | 0.0000 |
|  | 7PnB | 1722 | 134 | 33.797 | -84.912 | -87.192 | 4.559 | 0.0328 | 0.0416 |
|  | 8PnB | 1589 | 138 | 36.127 | -87.089 | -83.202 | 7.775 | 0.0053 | 0.0092 |
|  | 9PnB | 2352 | 150 | 35.532 | -109.741 | -103.343 | 12.796 | 0.0003 | 0.0008 |

* *p-*value adjusted>0.05, indicates passing of the neutrality test.

*J*: the total number of reads in the sample, *S*: the number of species in the sample, *θ*: fundamental biodiversity number, log(*L*_0_) is the log-likelihood of the observed sample, log(*L*_1_) is the log-likelihood predicted by the neutral model, and *q*-value and *p*-value are the values of the likelihood ratios, and *p-*value adjusted with multiple correlation correction procedure detailed in the section of material and methods.
