# Supplementary material for: Oral microbial community assembly under the influence of periodontitis
Source: PLoS One. 2017 Aug 16;12(8):e0182259. doi: 10.1371/journal.pone.0182259 (PMC5558961; doi:10.1371/journal.pone.0182259)
Supplement: S2 Table — (DOC) [file pone.0182259.s002.doc]

**S2 Table**. The result of the neutrality test using Etienne’s formula (Full results for all samples)

| Treatment | *ID* | *J* | *S* | *θ * | *m* | log(L_0_) | log(L_1_) | *q-value* | *p-value* | *p-value*  *Adjusted* |
| --- | --- | --- | --- | --- | --- | --- | --- | --- | --- | --- |
| Control | 24H2 | 775 | 38 | 8.236 | 0.99801 | -48.437 | -52.858 | 8.840 | 0.0029 | 0.0060 |
|  | 25H1 | 890 | 29 | 5.621 | 0.99998 | -42.105 | -51.499 | 18.789 | 0.0000 | 0.0000 |
|  | 25H2* | 537 | 37 | 8.815 | 0.99710 | -44.025 | -44.720 | 1.391 | 0.2383 | 0.2692 |
|  | 26H1 | 917 | 53 | 12.104 | 0.99818 | -58.628 | -61.161 | 5.066 | 0.0244 | 0.0346 |
|  | 27H1 | 741 | 75 | 20.661 | 0.99972 | -59.764 | -55.539 | 8.451 | 0.0036 | 0.0070 |
|  | 27H2 | 891 | 124 | 38.918 | 0.99981 | -63.339 | -58.654 | 9.370 | 0.0022 | 0.0046 |
|  | 28H1 | 1030 | 86 | 22.127 | 0.99995 | -64.608 | -66.682 | 4.148 | 0.0417 | 0.0530 |
|  | 28H2* | 687 | 75 | 21.273 | 0.99998 | -53.352 | -52.881 | 0.942 | 0.3319 | 0.3681 |
|  | 29H1 | 6126 | 123 | 21.747 | 0.99973 | -142.954 | -159.000 | 32.092 | 0.0000 | 0.0000 |
|  | 29H2 | 5567 | 66 | 10.383 | 0.99964 | -101.011 | -126.362 | 50.702 | 0.0000 | 0.0000 |
|  | 30H1 | 5294 | 68 | 10.963 | 0.99891 | -107.556 | -126.000 | 36.889 | 0.0000 | 0.0000 |
|  | 30H2 | 4922 | 71 | 11.696 | 0.99771 | -100.181 | -125.142 | 49.923 | 0.0000 | 0.0000 |
|  | 31H1 | 6810 | 77 | 12.086 | 0.98820 | -109.742 | -142.194 | 64.905 | 0.0000 | 0.0000 |
|  | 31H2 | 10130 | 54 | 7.425 | 0.99930 | -91.386 | -136.208 | 89.643 | 0.0000 | 0.0000 |
|  | 32H1 | 8670 | 65 | 9.544 | 0.99900 | -98.920 | -140.687 | 83.535 | 0.0000 | 0.0000 |
|  | 33H1 | 8366 | 75 | 11.292 | 0.99355 | -111.342 | -147.154 | 71.624 | 0.0000 | 0.0000 |
|  | 33H2 | 4575 | 74 | 12.537 | 0.98856 | -109.482 | -123.380 | 27.796 | 0.0000 | 0.0000 |
| BoP | 10PB | 3159 | 137 | 29.490 | 0.90150 | -119.216 | -121.671 | 4.910 | 0.0267 | 0.0370 |
|  | 11PB | 2764 | 92 | 18.174 | 0.99991 | -91.589 | -108.623 | 34.068 | 0.0000 | 0.0000 |
|  | 12PB* | 825 | 85 | 23.597 | 0.99973 | -60.078 | -59.020 | 2.114 | 0.1459 | 0.1679 |
|  | 13PB | 1210 | 125 | 34.802 | 0.99999 | -73.992 | -71.304 | 5.375 | 0.0204 | 0.0304 |
|  | 14PB | 3606 | 76 | 13.583 | 0.98883 | -89.486 | -116.044 | 53.115 | 0.0000 | 0.0000 |
|  | 15PB | 3211 | 86 | 16.153 | 0.99978 | -108.847 | -113.196 | 8.699 | 0.0032 | 0.0063 |
|  | 16PB | 1176 | 82 | 19.919 | 0.99993 | -64.286 | -71.837 | 15.103 | 0.0001 | 0.0003 |
|  | 17PB* | 1421 | 106 | 26.315 | 0.99875 | -78.995 | -79.284 | 0.578 | 0.4471 | 0.4785 |
|  | 18PB | 1116 | 128 | 38.435 | 0.83510 | -69.543 | -67.220 | 4.646 | 0.0311 | 0.0404 |
|  | 19PB | 1025 | 122 | 40.285 | 0.59478 | -68.244 | -64.203 | 8.082 | 0.0045 | 0.0083 |
|  | 1PB | 1788 | 97 | 21.880 | 0.99974 | -84.900 | -89.703 | 9.606 | 0.0019 | 0.0042 |
|  | 20PB | 1247 | 157 | 47.280 | 0.99947 | -79.697 | -69.501 | 20.392 | 0.0000 | 0.0000 |
|  | 21PB | 1487 | 101 | 24.357 | 0.99994 | -76.008 | -81.797 | 11.579 | 0.0007 | 0.0017 |
|  | 23PB | 947 | 100 | 28.022 | 0.99995 | -65.227 | -62.882 | 4.690 | 0.0303 | 0.0402 |
|  | 2PB | 1205 | 58 | 12.616 | 0.99957 | -60.290 | -69.201 | 17.823 | 0.0000 | 0.0001 |
|  | 3PB* | 2089 | 122 | 28.112 | 0.99982 | -97.807 | -97.918 | 0.222 | 0.6374 | 0.6480 |
|  | 4PB | 1157 | 109 | 29.358 | 0.99918 | -74.664 | -70.849 | 7.631 | 0.0057 | 0.0100 |
|  | 5PB | 1132 | 80 | 19.471 | 0.99971 | -66.765 | -70.658 | 7.787 | 0.0053 | 0.0094 |
|  | 6PB | 2334 | 80 | 15.948 | 0.99797 | -78.694 | -97.720 | 38.051 | 0.0000 | 0.0000 |
|  | 7PB* | 3423 | 165 | 36.898 | 0.80972 | -125.253 | -127.142 | 3.778 | 0.0519 | 0.0647 |
|  | 8PB | 4334 | 153 | 30.738 | 0.99974 | -131.383 | -142.460 | 22.155 | 0.0000 | 0.0000 |
|  | 9PB* | 3600 | 130 | 26.320 | 0.99974 | -127.906 | -129.013 | 2.214 | 0.1367 | 0.1635 |
| Non-BoP | 10PnB | 1532 | 138 | 36.552 | 0.99993 | -84.990 | -81.394 | 7.192 | 0.0073 | 0.0124 |
|  | 11PnB | 1929 | 91 | 19.672 | 0.99977 | -89.606 | -92.908 | 6.604 | 0.0102 | 0.0159 |
|  | 12PnB | 2451 | 89 | 17.960 | 0.99977 | -94.224 | -101.945 | 15.442 | 0.0001 | 0.0002 |
|  | 13PnB* | 1192 | 75 | 17.637 | 0.99989 | -73.193 | -72.103 | 2.179 | 0.1399 | 0.1641 |
|  | 14PnB | 3505 | 50 | 8.185 | 0.99976 | -81.462 | -98.063 | 33.202 | 0.0000 | 0.0000 |
|  | 15PnB | 3238 | 60 | 10.306 | 0.99964 | -83.407 | -102.737 | 38.660 | 0.0000 | 0.0000 |
|  | 16PnB | 1714 | 58 | 11.513 | 0.99971 | -63.167 | -80.567 | 34.800 | 0.0000 | 0.0000 |
|  | 17PnB | 1745 | 129 | 31.882 | 0.99754 | -91.234 | -88.826 | 4.817 | 0.0282 | 0.0382 |
|  | 18PnB* | 1147 | 99 | 25.834 | 0.99988 | -71.087 | -70.895 | 0.384 | 0.5354 | 0.5631 |
|  | 19PnB | 1271 | 85 | 20.326 | 0.99998 | -77.750 | -74.867 | 5.766 | 0.0163 | 0.0249 |
|  | 1PnB* | 1453 | 106 | 26.171 | 0.99993 | -80.265 | -80.192 | 0.146 | 0.7026 | 0.7026 |
|  | 20PnB | 1134 | 122 | 34.528 | 0.99987 | -74.453 | -68.770 | 11.367 | 0.0007 | 0.0018 |
|  | 21PnB* | 591 | 95 | 31.743 | 0.99995 | -47.409 | -46.262 | 2.294 | 0.1298 | 0.1584 |
|  | 23PnB* | 3569 | 131 | 26.571 | 0.99562 | -127.804 | -128.135 | 0.662 | 0.4159 | 0.4531 |
|  | 2PnB | 2062 | 83 | 17.233 | 0.99981 | -86.946 | -94.541 | 15.190 | 0.0001 | 0.0003 |
|  | 3PnB | 1077 | 125 | 36.365 | 0.99996 | -71.265 | -66.454 | 9.622 | 0.0019 | 0.0042 |
|  | 4PnB | 833 | 59 | 14.329 | 0.99968 | -62.012 | -58.633 | 6.758 | 0.0093 | 0.0150 |
|  | 5PnB* | 1824 | 126 | 30.621 | 0.99991 | -90.962 | -91.094 | 0.265 | 0.6068 | 0.6274 |
|  | 6PnB | 4172 | 118 | 22.459 | 0.99995 | -115.159 | -134.710 | 39.101 | 0.0000 | 0.0000 |
|  | 7PnB | 1722 | 134 | 33.987 | 0.98637 | -84.983 | -87.597 | 5.228 | 0.0222 | 0.0323 |
|  | 8PnB | 1589 | 138 | 36.221 | 0.99137 | -87.159 | -83.656 | 7.006 | 0.0081 | 0.0134 |
|  | 9PnB | 2352 | 150 | 35.572 | 0.99987 | -109.742 | -104.329 | 10.826 | 0.0010 | 0.0023 |

* *p-*value adjusted>0.05, indicates passing of the neutrality test.

*J*: the total number of reads in the sample, *S*: the number of species in the sample, *θ*: fundamental biodiversity number, *m*: immigration probability, log(*L*_0_) is the log-likelihood of the observed sample, log(*L*_1_) is the log-likelihood predicted by the neutral model, and *q*-value and *p*-value are the values of the likelihood ratios, and *p-*value adjusted with multiple correlation correction procedure detailed in the section of material and methods.
