# Supplementary material for: Oral microbial community assembly under the influence of periodontitis
Source: PLoS One. 2017 Aug 16;12(8):e0182259. doi: 10.1371/journal.pone.0182259 (PMC5558961; doi:10.1371/journal.pone.0182259)
Supplement: S3 Table — (DOC) [file pone.0182259.s003.doc]

**S3 Table**. The detailed results from the comparison of Ewens and Etienne sampling formulae*

| Treatment | ID | *J* | *S* | *θ_1_* | log(*L*_1_) | *θ_2_* | *m* | log(*L*_2_) | *q*-value | *p*-value |
| --- | --- | --- | --- | --- | --- | --- | --- | --- | --- | --- |
| Healthy | 24H2 | 775 | 38 | 8.231 | -52.651 | 8.236 | 0.99801 | -52.858 | 0.207 | 0.649 |
|  | 25H1 | 890 | 29 | 5.617 | -50.813 | 5.621 | 0.99998 | -51.499 | 0.686 | 0.408 |
|  | 25H2* | 537 | 37 | 8.856 | -44.603 | 8.815 | 0.9971 | -44.72 | 0.117 | 0.732 |
|  | 26H1 | 917 | 53 | 12.092 | -60.922 | 12.104 | 0.99818 | -61.161 | 0.239 | 0.625 |
|  | 27H1 | 741 | 75 | 20.653 | -55.181 | 20.661 | 0.99972 | -55.539 | 0.358 | 0.550 |
|  | 27H2 | 891 | 124 | 38.921 | -57.362 | 38.918 | 0.99981 | -58.654 | 1.292 | 0.256 |
|  | 28H1 | 1030 | 86 | 22.147 | -66.577 | 22.127 | 0.99995 | -66.682 | 0.105 | 0.746 |
|  | 28H2* | 687 | 75 | 21.25 | -52.651 | 21.273 | 0.99998 | -52.881 | 0.23 | 0.632 |
|  | 29H1 | 6126 | 123 | 21.693 | -158.78 | 21.747 | 0.99973 | -159 | 0.22 | 0.639 |
|  | 29H2 | 5567 | 66 | 10.425 | -125.872 | 10.383 | 0.99964 | -126.362 | 0.49 | 0.484 |
|  | 30H1 | 5294 | 68 | 10.909 | -125.47 | 10.963 | 0.99891 | -126 | 0.53 | 0.467 |
|  | 30H2 | 4922 | 71 | 11.656 | -122.945 | 11.696 | 0.99771 | -125.142 | 2.197 | 0.138 |
|  | 31H1 | 6810 | 77 | 12.071 | -141.761 | 12.086 | 0.9882 | -142.194 | 0.433 | 0.511 |
|  | 31H2 | 10130 | 54 | 7.407 | -135.914 | 7.425 | 0.9993 | -136.208 | 0.294 | 0.588 |
|  | 32H1 | 8670 | 65 | 9.453 | -143.776 | 9.544 | 0.999 | -140.687 | 3.089 | 0.079 |
|  | 33H1 | 8366 | 75 | 11.267 | -148.781 | 11.292 | 0.99355 | -147.154 | 1.627 | 0.202 |
|  | 33H2 | 4575 | 74 | 12.434 | -123.95 | 12.537 | 0.98856 | -123.38 | 0.57 | 0.450 |
| BoP | 10PB | 3159 | 137 | 29.056 | -121.024 | 29.49 | 0.9015 | -121.671 | 0.647 | 0.421 |
|  | 11PB | 2764 | 92 | 18.19 | -108.174 | 18.174 | 0.99991 | -108.623 | 0.449 | 0.503 |
|  | 12PB* | 825 | 85 | 23.587 | -58.518 | 23.597 | 0.99973 | -59.02 | 0.502 | 0.479 |
|  | 13PB | 1210 | 125 | 34.811 | -70.834 | 34.802 | 0.99999 | -71.304 | 0.47 | 0.493 |
|  | 14PB | 3606 | 76 | 13.503 | -113.892 | 13.583 | 0.98883 | -116.044 | 2.152 | 0.142 |
|  | 15PB | 3211 | 86 | 16.137 | -112.691 | 16.153 | 0.99978 | -113.196 | 0.505 | 0.477 |
|  | 16PB | 1176 | 82 | 19.898 | -71.445 | 19.919 | 0.99993 | -71.837 | 0.392 | 0.531 |
|  | 17PB* | 1421 | 106 | 26.333 | -78.677 | 26.315 | 0.99875 | -79.284 | 0.607 | 0.436 |
|  | 18PB | 1116 | 128 | 37.107 | -66.873 | 38.435 | 0.8351 | -67.22 | 0.347 | 0.556 |
|  | 19PB | 1025 | 122 | 35.88 | -63.739 | 40.285 | 0.59478 | -64.203 | 0.464 | 0.496 |
|  | 1PB | 1788 | 97 | 21.849 | -88.821 | 21.88 | 0.99974 | -89.703 | 0.882 | 0.348 |
|  | 20PB | 1247 | 157 | 47.296 | -69.705 | 47.28 | 0.99947 | -69.501 | 0.204 | 0.652 |
|  | 21PB | 1487 | 101 | 24.344 | -81.043 | 24.357 | 0.99994 | -81.797 | 0.754 | 0.385 |
|  | 23PB | 947 | 100 | 28.041 | -62.252 | 28.022 | 0.99995 | -62.882 | 0.63 | 0.427 |
|  | 2PB | 1205 | 58 | 12.573 | -69.172 | 12.616 | 0.99957 | -69.201 | 0.029 | 0.865 |
|  | 3PB* | 2089 | 122 | 28.116 | -97.035 | 28.112 | 0.99982 | -97.918 | 0.883 | 0.347 |
|  | 4PB | 1157 | 109 | 29.326 | -70.259 | 29.358 | 0.99918 | -70.849 | 0.59 | 0.442 |
|  | 5PB | 1132 | 80 | 19.492 | -69.87 | 19.471 | 0.99971 | -70.658 | 0.788 | 0.375 |
|  | 6PB | 2334 | 80 | 15.916 | -98.006 | 15.948 | 0.99797 | -97.72 | 0.286 | 0.593 |
|  | 7PB | 3423 | 165 | 36.043 | -127.578 | 36.898 | 0.80972 | -127.142 | 0.436 | 0.509 |
|  | 8PB | 4334 | 153 | 30.781 | -141.395 | 30.738 | 0.99974 | -142.46 | 1.065 | 0.302 |
|  | 9PB* | 3600 | 130 | 26.284 | -128.182 | 26.32 | 0.99974 | -129.013 | 0.831 | 0.362 |
| Non-BoP | 10PnB | 1532 | 138 | 36.589 | -81.142 | 36.552 | 0.99993 | -81.394 | 0.252 | 0.616 |
|  | 11PnB | 1929 | 91 | 19.698 | -92.015 | 19.672 | 0.99977 | -92.908 | 0.893 | 0.345 |
|  | 12PnB | 2451 | 89 | 17.979 | -101.631 | 17.96 | 0.99977 | -101.945 | 0.314 | 0.575 |
|  | 13PnB* | 1192 | 75 | 17.616 | -71.624 | 17.637 | 0.99989 | -72.103 | 0.479 | 0.489 |
|  | 14PnB | 3505 | 50 | 8.16 | -96.841 | 8.185 | 0.99976 | -98.063 | 1.222 | 0.269 |
|  | 15PnB | 3238 | 60 | 10.348 | -103.375 | 10.306 | 0.99964 | -102.737 | 0.638 | 0.424 |
|  | 16PnB | 1714 | 58 | 11.468 | -80.41 | 11.513 | 0.99971 | -80.567 | 0.157 | 0.692 |
|  | 17PnB | 1745 | 129 | 31.988 | -88.156 | 31.882 | 0.99754 | -88.826 | 0.67 | 0.413 |
|  | 18PnB* | 1147 | 99 | 25.812 | -70.521 | 25.834 | 0.99988 | -70.895 | 0.374 | 0.541 |
|  | 19PnB | 1271 | 85 | 20.364 | -74.665 | 20.326 | 0.99998 | -74.867 | 0.202 | 0.653 |
|  | 1PnB | 1453 | 106 | 26.144 | -80.596 | 26.171 | 0.99993 | -80.192 | 0.404 | 0.525 |
|  | 20PnB | 1134 | 122 | 34.494 | -67.846 | 34.528 | 0.99987 | -68.77 | 0.924 | 0.336 |
|  | 21PnB* | 591 | 95 | 31.763 | -46.374 | 31.743 | 0.99995 | -46.262 | 0.112 | 0.738 |
|  | 23PnB | 3569 | 131 | 26.596 | -127.626 | 26.571 | 0.99562 | -128.135 | 0.509 | 0.476 |
|  | 2PnB | 2062 | 83 | 17.207 | -93.606 | 17.233 | 0.99981 | -94.541 | 0.935 | 0.334 |
|  | 3PnB | 1077 | 125 | 36.402 | -65.917 | 36.365 | 0.99996 | -66.454 | 0.537 | 0.464 |
|  | 4PnB | 833 | 59 | 14.343 | -58.605 | 14.329 | 0.99968 | -58.633 | 0.028 | 0.867 |
|  | 5PnB* | 1824 | 126 | 30.571 | -90.718 | 30.621 | 0.99991 | -91.094 | 0.376 | 0.540 |
|  | 6PnB | 4172 | 118 | 22.469 | -133.872 | 22.459 | 0.99995 | -134.71 | 0.838 | 0.360 |
|  | 7PnB | 1722 | 134 | 33.797 | -87.192 | 33.987 | 0.98637 | -87.597 | 0.405 | 0.525 |
|  | 8PnB | 1589 | 138 | 36.127 | -83.202 | 36.221 | 0.99137 | -83.656 | 0.454 | 0.500 |
|  | 9PnB | 2352 | 150 | 35.532 | -103.343 | 35.572 | 0.99987 | -104.329 | 0.986 | 0.321 |

* *p*>0.05, indicates samples that passed the neutrality test.

*J*: the total number of reads in the sample, *S*: the number of species in the sample, *θ*: fundamental biodiversity, m: immigration probability, log(*L*_1_) is the log-likelihood from using Ewen’s formula, log(*L*_2_) is the log-likelihood from using Etienne formulae, and *q*-value and *p*-value are the values of the likelihood ratios.
