# Supplementary material for: Oral microbial community assembly under the influence of periodontitis
Source: PLoS One. 2017 Aug 16;12(8):e0182259. doi: 10.1371/journal.pone.0182259 (PMC5558961; doi:10.1371/journal.pone.0182259)
Supplement: S4 Table — (DOC) [file pone.0182259.s004.doc]

**S4 Table**. The result of the neutrality test using Etienne formula with 1000 times of simulations*

| Group | *ID* | *J* | *S* | *θ * | *m* | log(L_0_) | log(L_1_) | *q-value* | *p-value* | *p-value*  *Adjusted* |
| --- | --- | --- | --- | --- | --- | --- | --- | --- | --- | --- |
| Healthy | 24H2 | 775 | 38 | 8.236 | 0.99801 | -48.437 | -53.292 | 9.710 | 0.0018 | 0.0038 |
|  | 25H1 | 890 | 29 | 5.621 | 0.99998 | -42.105 | -51.007 | 17.803 | 0.0000 | 0.0000 |
|  | 25H2* | 537 | 37 | 8.815 | 0.99710 | -44.025 | -45.171 | 2.293 | 0.1300 | 0.1496 |
|  | 26H1 | 917 | 53 | 12.104 | 0.99818 | -58.628 | -61.199 | 5.142 | 0.0234 | 0.0317 |
|  | 27H1 | 741 | 75 | 20.661 | 0.99972 | -59.764 | -55.241 | 9.046 | 0.0026 | 0.0048 |
|  | 27H2 | 891 | 124 | 38.918 | 0.99981 | -63.339 | -58.648 | 9.383 | 0.0022 | 0.0043 |
|  | 28H1 | 1030 | 86 | 22.127 | 0.99995 | -64.608 | -67.145 | 5.074 | 0.0243 | 0.0322 |
|  | 28H2* | 687 | 75 | 21.273 | 0.99998 | -53.352 | -53.006 | 0.692 | 0.4054 | 0.4191 |
|  | 29H1 | 6126 | 123 | 21.747 | 0.99973 | -142.954 | -160.043 | 34.178 | 0.0000 | 0.0000 |
|  | 29H2 | 5567 | 66 | 10.383 | 0.99964 | -101.011 | -126.552 | 51.082 | 0.0000 | 0.0000 |
|  | 30H1 | 5294 | 68 | 10.963 | 0.99891 | -107.556 | -125.796 | 36.480 | 0.0000 | 0.0000 |
|  | 30H2 | 4922 | 71 | 11.696 | 0.99771 | -100.181 | -124.840 | 49.318 | 0.0000 | 0.0000 |
|  | 31H1 | 6810 | 77 | 12.086 | 0.98820 | -109.742 | -142.284 | 65.086 | 0.0000 | 0.0000 |
|  | 31H2 | 10130 | 54 | 7.425 | 0.99930 | -91.386 | -135.153 | 87.533 | 0.0000 | 0.0000 |
|  | 32H1 | 8670 | 65 | 9.544 | 0.99900 | -98.920 | -141.262 | 84.683 | 0.0000 | 0.0000 |
|  | 33H1 | 8366 | 75 | 11.292 | 0.99355 | -111.342 | -148.344 | 74.005 | 0.0000 | 0.0000 |
|  | 33H2 | 4575 | 74 | 12.537 | 0.98856 | -109.482 | -123.566 | 28.168 | 0.0000 | 0.0000 |
| PoB | 10PB | 3159 | 137 | 29.490 | 0.90150 | -119.216 | -121.962 | 5.493 | 0.0191 | 0.0299 |
|  | 11PB | 2764 | 92 | 18.174 | 0.99991 | -91.589 | -108.249 | 33.320 | 0.0000 | 0.0000 |
|  | 12PB* | 825 | 85 | 23.597 | 0.99973 | -60.078 | -58.851 | 2.453 | 0.1173 | 0.1403 |
|  | 13PB | 1210 | 125 | 34.802 | 0.99999 | -73.992 | -71.423 | 5.138 | 0.0234 | 0.0317 |
|  | 14PB | 3606 | 76 | 13.583 | 0.98883 | -89.486 | -115.341 | 51.709 | 0.0000 | 0.0000 |
|  | 15PB | 3211 | 86 | 16.153 | 0.99978 | -108.847 | -114.013 | 10.333 | 0.0013 | 0.0029 |
|  | 16PB | 1176 | 82 | 19.919 | 0.99993 | -64.286 | -71.799 | 15.026 | 0.0001 | 0.0003 |
|  | 17PB* | 1421 | 106 | 26.315 | 0.99875 | -78.995 | -79.602 | 1.214 | 0.2706 | 0.3001 |
|  | 18PB* | 1116 | 128 | 38.435 | 0.83510 | -69.543 | -67.858 | 3.369 | 0.0664 | 0.0810 |
|  | 19PB | 1025 | 122 | 40.285 | 0.59478 | -68.244 | -63.550 | 9.387 | 0.0022 | 0.0043 |
|  | 1PB | 1788 | 97 | 21.880 | 0.99974 | -84.900 | -89.758 | 9.717 | 0.0018 | 0.0038 |
|  | 20PB | 1247 | 157 | 47.280 | 0.99947 | -79.697 | -69.782 | 19.830 | 0.0000 | 0.0000 |
|  | 21PB | 1487 | 101 | 24.357 | 0.99994 | -76.008 | -81.792 | 11.568 | 0.0007 | 0.0017 |
|  | 23PB | 947 | 100 | 28.022 | 0.99995 | -65.227 | -62.614 | 5.226 | 0.0223 | 0.0316 |
|  | 2PB | 1205 | 58 | 12.616 | 0.99957 | -60.290 | -70.728 | 20.877 | 0.0000 | 0.0000 |
|  | 3PB* | 2089 | 122 | 28.112 | 0.99982 | -97.807 | -98.374 | 1.133 | 0.2870 | 0.3109 |
|  | 4PB | 1157 | 109 | 29.358 | 0.99918 | -74.664 | -70.651 | 8.027 | 0.0046 | 0.0083 |
|  | 5PB | 1132 | 80 | 19.471 | 0.99971 | -66.765 | -70.218 | 6.907 | 0.0086 | 0.0142 |
|  | 6PB | 2334 | 80 | 15.948 | 0.99797 | -78.694 | -97.712 | 38.036 | 0.0000 | 0.0000 |
|  | 7PB | 3423 | 165 | 36.898 | 0.80972 | -125.253 | -127.888 | 5.270 | 0.0217 | 0.0316 |
|  | 8PB | 4334 | 153 | 30.738 | 0.99974 | -131.383 | -142.576 | 22.388 | 0.0000 | 0.0000 |
|  | 9PB* | 3600 | 130 | 26.320 | 0.99974 | -127.906 | -129.745 | 3.679 | 0.0551 | 0.0686 |
| Non-BoP | 10PnB | 1532 | 138 | 36.552 | 0.99993 | -84.990 | -81.819 | 6.343 | 0.0118 | 0.0189 |
|  | 11PnB | 1929 | 91 | 19.672 | 0.99977 | -89.606 | -92.230 | 5.246 | 0.0220 | 0.0316 |
|  | 12PnB | 2451 | 89 | 17.960 | 0.99977 | -94.224 | -102.908 | 17.368 | 0.0000 | 0.0000 |
|  | 13PnB* | 1192 | 75 | 17.637 | 0.99989 | -73.193 | -72.150 | 2.086 | 0.1486 | 0.1679 |
|  | 14PnB | 3505 | 50 | 8.185 | 0.99976 | -81.462 | -97.985 | 33.046 | 0.0000 | 0.0000 |
|  | 15PnB | 3238 | 60 | 10.306 | 0.99964 | -83.407 | -101.808 | 36.803 | 0.0000 | 0.0000 |
|  | 16PnB | 1714 | 58 | 11.513 | 0.99971 | -63.167 | -80.749 | 35.164 | 0.0000 | 0.0000 |
|  | 17PnB | 1745 | 129 | 31.882 | 0.99754 | -91.234 | -88.806 | 4.858 | 0.0275 | 0.0357 |
|  | 18PnB* | 1147 | 99 | 25.834 | 0.99988 | -71.087 | -70.916 | 0.342 | 0.5584 | 0.5677 |
|  | 19PnB | 1271 | 85 | 20.326 | 0.99998 | -77.750 | -75.106 | 5.289 | 0.0215 | 0.0316 |
|  | 1PnB* | 1453 | 106 | 26.171 | 0.99993 | -80.265 | -80.824 | 1.117 | 0.2905 | 0.3109 |
|  | 20PnB | 1134 | 122 | 34.528 | 0.99987 | -74.453 | -68.653 | 11.601 | 0.0007 | 0.0017 |
|  | 21PnB* | 591 | 95 | 31.743 | 0.99995 | -47.409 | -46.244 | 2.330 | 0.1269 | 0.1489 |
|  | 23PnB* | 3569 | 131 | 26.571 | 0.99562 | -127.804 | -127.800 | 0.008 | 0.9268 | 0.9268 |
|  | 2PnB | 2062 | 83 | 17.233 | 0.99981 | -86.946 | -94.039 | 14.186 | 0.0002 | 0.0005 |
|  | 3PnB | 1077 | 125 | 36.365 | 0.99996 | -71.265 | -66.679 | 9.171 | 0.0025 | 0.0048 |
|  | 4PnB | 833 | 59 | 14.329 | 0.99968 | -62.012 | -59.607 | 4.810 | 0.0283 | 0.0360 |
|  | 5PnB* | 1824 | 126 | 30.621 | 0.99991 | -90.962 | -90.610 | 0.703 | 0.4017 | 0.4191 |
|  | 6PnB | 4172 | 118 | 22.459 | 0.99995 | -115.159 | -134.928 | 39.537 | 0.0000 | 0.0000 |
|  | 7PnB | 1722 | 134 | 33.987 | 0.98637 | -84.983 | -88.572 | 7.178 | 0.0074 | 0.0125 |
|  | 8PnB | 1589 | 138 | 36.221 | 0.99137 | -87.159 | -83.456 | 7.405 | 0.0065 | 0.0113 |
|  | 9PnB | 2352 | 150 | 35.572 | 0.99987 | -109.742 | -104.443 | 10.597 | 0.0011 | 0.0026 |

* *p*>0.05, indicates samples that passed the neutrality test.

*J*: the total number of reads (individuals) in the sample, *S*: the number of species in the sample, θ: fundamental biodiversity, *m*: immigration probability, log(L*_0_*) is the log-likelihood of the observed sample, log(*L_1_*) is the log-likelihood predicted by the neutral model, and *q*-value and *p*-value are the values of the likelihood ratios.
